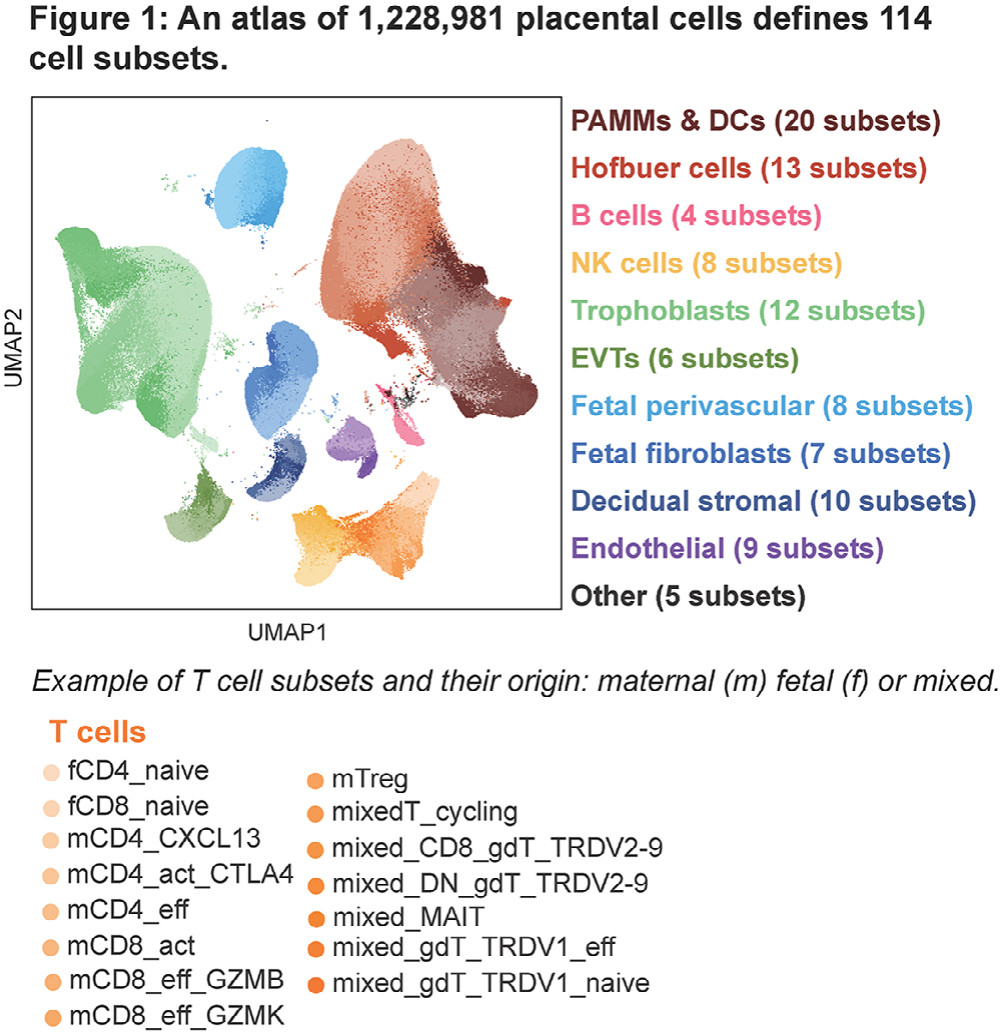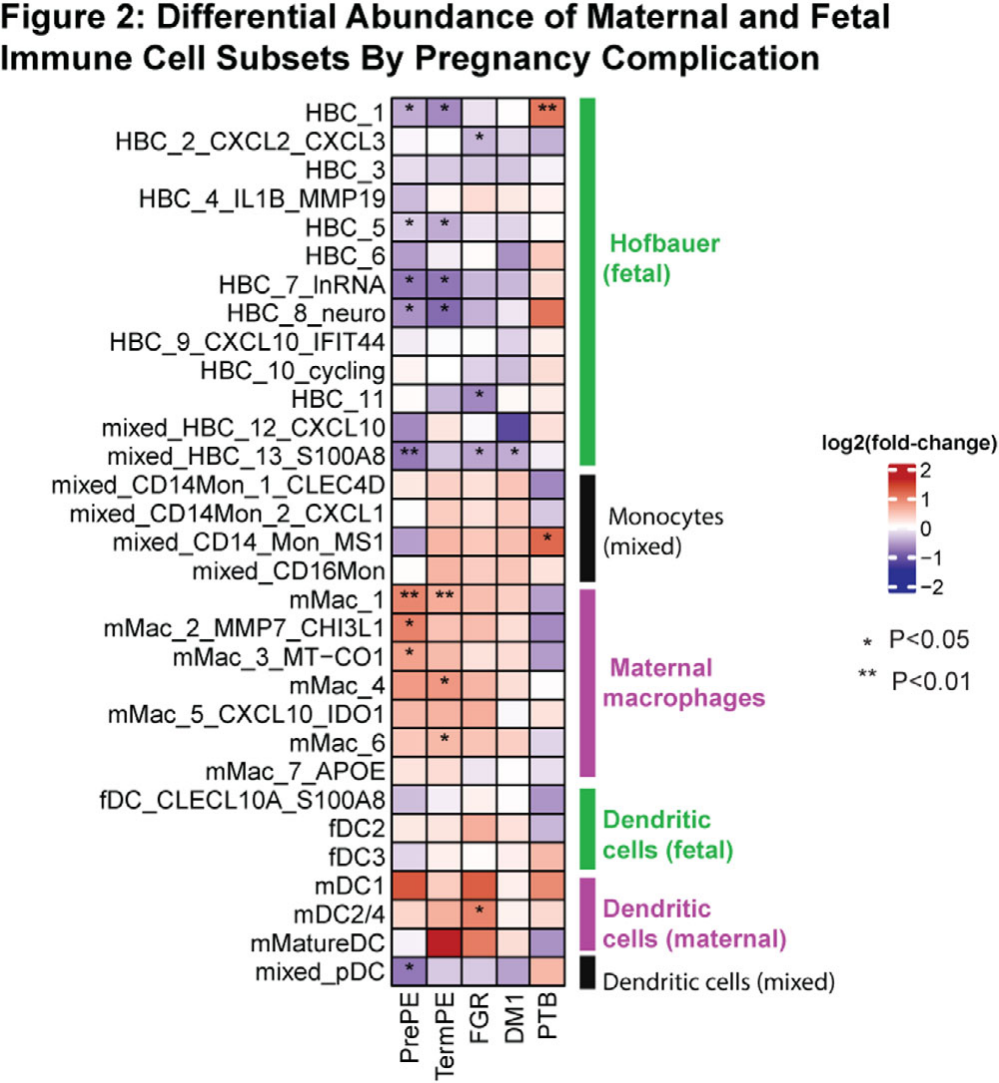# Oral Concurrent Session 7 – Basic and Translational Science: Friday, February 13, 2026 8:00 AM – 10:00 AM

**DOI:** 10.1002/pmf2.70155

**Published:** 2026-02-09

**8:00 AM  –  8:15 AM**

## 74 | **Proteomic Profile of Placenta Accreta Spectrum Highlighting Dysregulation and Paracrine Effects Adjacent to Cesarean Scar**

Jonathan L. Hecht^1^; Anna M. Modest^1^; Uma S. Deshmukh^1^; Saira L. Salahuddin^1^; Simon T. Dillon^1^; Yalda Afshar^2^; S Ananth Karumanchi^3^; Towia A. Libermann^1^; Scott A. Shainker^4^

^1^Beth Israel Deaconess Medical Center, Boston, MA; ^2^David Geffen School of Medicine at University of California, Los Angeles (UCLA), Los Angeles, CA; ^3^Cedars‐Sinai Medical Center, eCedars‐Sinai Medical Center,, CA; ^4^Department of Obstetrics and Gynecology, Beth Israel Deaconess Medical Center, Harvard Medical School, Boston, MA

**Objective**: Placenta accreta spectrum (PAS) represents abnormal placental adherence and uterine wall infiltration. While deep infiltration (FIGO grade 3) is mediated by cesarean scar dehiscence, the mechanism of adherence beyond the scar remains elusive. We hypothesize that the microenvironment in the area of scar is propagated along the utero‐placental interface.

**Study Design**: Fresh samples were obtained along the uteroplacental interface in hysterectomies for PAS; adjacent to scar (PAS‐deep) and in areas of placental adherence distant from the scar (PAS‐ superficial). Placental bed punch biopsies were obtained from non‐PAS hysterectomies with placenta previa (non‐PAS controls). Proteomics 11k SomaScan v5.0 assay data was analysed for placenta enriched proteins. Differences were reported using t‐test, *p* < 0.05 was considered significant.

**Results**: Samples included 12 PAS cases with paired tissue from PAS‐deep and PAS‐superficial areas, and 9 non‐PAS controls. There were significant changes in protein abundance between PAS‐deep and non‐PAS controls (Figure 1). PSG3, ADAM12, and MAGEA8 were differentially expressed in areas of PAS‐deep compared to PAS‐superficial areas (all *p* ≤ 0.035) (Figure 2). The findings suggest over‐activation of placental implantation along the utero‐placental interface in a gradient extending from the scar. There were significant differences in expression of PSG1, PSG9, PSG3, ISM2, ADAM12, and LUM in PAS‐invasive compared to non‐PAS samples. (all *p* ≤ 0.002)

**Conclusion**: Proteomics analysis shows a gradient of proteins related to implantation from areas of deep penetration to superficial adherence, supporting a model in which PAS arises from abnormal implantation adjacent to cesarean scar dehiscence. Proteins involving angiogenesis, extracellular matrix remodeling, trophoblast invasion and immune modulation were discovered, potentially highlighting their role in pathogenesis of PAS. These finding suggest paracrine signaling may extend the uterine scar leading to a gradient of placental adherence.

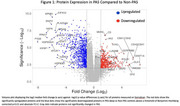

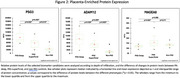

**8:15 AM  –  8:30 AM**

## 75 | Unraveling the Pathogenesis of Common Pregnancy Complications: an Atlas of 1.2 Million Placental Cells

Paola A. Lopez‐Zapana^1^; Rachelly Normand^2^; Daehee Han^3^; Courtney Ambrose^4^; Roya Best^5^; Zhaojing Liu^1^; Elizabeth Tuttle^5^; Christopher Stueber^5^; Olyvia J. Jasset^1^; Laura Ibanez‐Pintor^1^; Lydia L. Shook^6^; Douglas A. Lauffenburger^7^; Alexandra‐Chloé Villani^8^; Andrea G. Edlow^9^

^1^Vincent Center for Reproductive Biology, Massachusetts General Hospital, Boston, MA; ^2^Massachusetts General Hospital, Massachusetts General Hospital, MA; ^3^Vincent Center for Reproductive Biology, Massachusetts General Hospital; Department of Obstetrics and Gynecology, MassGeneral Brigham; Division of Maternal‐Fetal Medicine; Harvard Medical School, Boston, MA; ^4^Center for Immunology and Inflammatory Diseases, Department of Medicine and Krantz Family Center for Cancer Research, Massachusetts General Hospital, Boston, MA; ^5^Massachusetts General Hospital, Boston, MA; ^6^Department of Obstetrics and Gynecology, MassGeneral Brigham; Division of Maternal‐Fetal Medicine; Harvard Medical School, Massachusetts General Hospital/Boston, MA; ^7^Department of Biological Engineering, Massachusetts Institute of Technology, Cambridge, MA; ^8^Center for Immunology and Inflammatory Diseases, Krantz Family Center for Cancer Research, Massachusetts General Hospital, Boston, MA; ^9^Department of Obstetrics and Gynecology, MassGeneral Brigham; Division of Maternal‐Fetal Medicine; Harvard Medical School, Boston, MA

**Objective**: Precise molecular and cellular phenotyping of the placenta in health and disease is needed to develop effective targeted therapies. Here we examined placental cellular programs and biological functions across 4 immune‐mediated pregnancy complications.

**Study Design**: Maternal and fetal cell populations were profiled with 10x Genomics scRNA‐seq in 59 placenta samples (*N* = 37 cases: fetal growth restriction [FGR, *n* = 9], preterm preeclampsia [prePE, *n* = 10], term preeclampsia [termPE, *n* = 8], and spontaneous preterm birth [SPTB, *n* = 10], and *N* = 22 controls from healthy term pregnancies [labor *n* = 9, no labor *n* = 9], or preterm unlabored controls [vasa previa, *n* = 4]). Differentially expressed genes in case vs control subclusters were identified and pathways analysis (Ingenuity) was performed.

**Results**: Sequencing 1,228,981 placental cells identified 114 unique cell populations from 16 parent lineages. Specific cell subsets were implicated in each pregnancy complication (Figure 1A). Fetal and maternal endothelial cells, maternal macrophages (mMacs), and a villous cytotrophoblast (VCT) subset were key in prePE but not termPE; fetal fibroblasts, decidual stromal cells, mMacs, and novel Hofbauer cell (HBC) populations were uniquely implicated in SPTB (Figure 1A). Shared and distinct gene programs were identified, with biological functions (Figure 1B) and canonical signaling pathways (Figure 2) dysregulated in particular cell types and pregnancy complications. Representative disease pathogenesis insights include: reduced angiogenesis in fetal endothelial cells and increased inflammatory mMac signaling in FGR; upregulation of interferon and metabolic signaling in mMacs and of angiogenesis in syncytiotrophoblasts (SCT) and VCT in prePE; but downregulation of angiogenesis in VCT and SCT with increased interferon and fatty acid signaling in HBCs in termPE.

**Conclusion**: This comprehensive placental single cell atlas identified condition‐ and cell‐specific gene programs underlying common pregnancy complications. Targeting these programs may enable precision strategies to improve maternal and fetal outcomes.

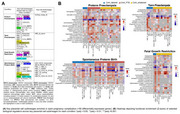

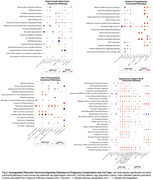

**8:30 AM  –  8:45 AM**

## 76 | **Uterine** Collagen **Gradients for Placenta Accreta Prevention Utilizing Second Harmonic Imaging Microscopy**

Sohum C. Shah^1^; Haley Marks^2^; Guadalupe Martinez^3^; Jakub Staniczek^4^; Alex Kot^2^; Roya Bagheri^5^; Deborah Krakow^6^; Yalda Afshar^6^

^1^Department of Obstetrics and Gynecology, David Geffen School of Medicine at University of California, Los Angeles (UCLA), UCLA, CA; ^2^University of California, Los Angeles (UCLA), UCLA, CA; ^3^University of California, Los Angeles (UCLA), Los Angeles, CA; ^4^Śląski Uniwersytet Medyczny w Katowicach, Katowice, Slaskie; ^5^University of California, Los Angeles (UCLA), UCLA Health, CA; ^6^David Geffen School of Medicine at University of California, Los Angeles (UCLA), Los Angeles, CA

**Objective**: Our previous data demonstrated aberrant type I collagen architecture at the site of placental adherence in PAS. Despite the clinical significance of PAS, prevention strategies are limited, with cesarean scar optimization representing an unmet clinical need. PAS results from abnormal trophoblast adherence at a uterine scar. We developed a novel second‐harmonic generation (SHG) imaging protocol to quantify collagen architecture in collagen‐deficient and wild‐type (WT) murine uteri with and without PAS, establishing the foundation for collagen‐based PAS prevention therapies at the cesarean scar.

**Study Design**: Type I collagen mutant mice were utilized (Col1a1+/− and Col1a1aga2 heterozygous) and compared to WT controls. Uteri and cervix were processed for en bloc SHG imaging followed by 20× sectional analysis. Blinded quantitative analysis assessed collagen density (fibrillar:background intensity ratio) across the cervix, proximal, and distal uterine regions. Statistical analysis used Mann‐Whitney U test with significance set at *p* < 0.05 and two‐way ANOVA with significance set at *p* < 0.05.

**Results**: WT demonstrated 3.2x higher collagen density versus mutants (mean ratio 2.85 ± 0.34 vs 0.89 ± 0.21, *p* < 0.001 (Figure 1). Regional analysis revealed a distinct anatomical gradient: cervix(3.1 ± 0.4), proximal horn(2.2 ± 0.3), distal horn (1.8 ± 0.2). Collagen organization differed markedly, with WT showing dense reticular networks versus sparse fibers in mutants (Figure 2). The cell‐cell adhesion molecule and extracellular matrix component, E‐cadherin, co‐localized with Type I collagen. PAS mice demonstrate disturbed collagen organization at the decidual‐placental border.

**Conclusion**: This validated SHG protocol enables precise collagen quantification and reveals critical regional variations in uterine architecture. This directly informs collagen‐restoration strategies for PAS prevention, with utero‐cervical junction targeting representing the highest‐yield intervention site. Translation to mouse PAS models and human cesarean scar assessment represents the immediate next step.

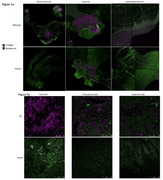

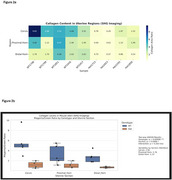

**8:45 AM  –  9:00 AM**

## 77 | **Urinary** Lipidomic **Profiling Associated With PM2.5 Exposure During Pregnancy**

Sunwha Park^1^; Minki Shim^2^; Young‐Ah You^3^; Young Min Hur^4^; Yoon‐Young Go^3^; Mi Hye Park^4^; Young‐Han Kim^5^; Sung Hun Na^6^; Geum Joon Cho^7^; Jin‐Gon Bae^8^; Soo‐Jeong Lee^9^; Dong‐Kyu Lee^2^; Young Ju Kim^4^

^1^Ewha Womans University Hospital, Ewha Womans University Hospital/Seoul, Seoul‐t'ukpyolsi; ^2^College of Pharmacy, Chung‐Ang University, Seoul, Seoul‐t'ukpyolsi; ^3^Ewha Medical Research Institute, Ewha Womans University, Seoul, Seoul‐t'ukpyolsi; ^4^Ewha Womans University Hospital, Seoul, Seoul‐t'ukpyolsi; ^5^Department of Obstetrics and Gynecology, Yonsei University College of Medicine, Department of Obstetrics and Gynecology, Yonsei University College of Medicine, Seoul‐t'ukpyolsi; ^6^Department of Obstetrics and Gynecology, Kangwon National University, School of Medicine, Department of Obstetrics and Gynecology, Kangwon National University, School of Medicine, Kangwon‐do; ^7^College of Medicine, Korea University, Seoul, Seoul‐t'ukpyolsi; ^8^Department of Obstetrics and Gynecology, Keimyung University, School of Medicine, Department of Obstetrics and Gynecology, Keimyung University, School of Medicine, Taegu‐jikhalsi; ^9^University of Ulsan College of Medicine, Ulsan, Ulsan‐gwangyoksi

**Objective**: To evaluate urinary lipidomic changes associated with PM2.5 exposure during pregnancy and explore their potential link to adverse pregnancy outcomes.

**Study Design**: We conducted a prospective multicenter cohort study of singleton pregnancies in South Korea (2021–2023). Indoor PM2.5 was measured using AirGuard K® monitors installed in participants’ homes; outdoor PM2.5 data were obtained from a national monitoring network. Personal exposure was estimated using time‐weighted average models incorporating time‐activity patterns. Among 1077 monitored women, 30 with low exposure (< 3 µg/m^3^) and 30 with high exposure (>15 µg/m^3^) were selected for urinary lipidomic profiling. Urinary lipids were extracted using modified Folch's method and analyzed by LC‐HRMS. A total of 117 lipid species across 7 subclasses were identified. Statistical analysis included PLS‐DA and univariate t‐tests. Clinical correlations were assessed using Pearson's correlation analysis.

**Results**: Multivariate analysis demonstrated clear separation of exposure groups. Sixty‐two lipids showed significant differences (*p* < 0.001), notably decreased phosphatidylethanolamines (PE) and saturated triacylglycerols (TG) in the high‐exposure group, with selective increases in unsaturated TGs (e.g., TG 52:2, TG 56:3). These lipids correlated with PM2.5 levels and oxidative stress markers (e.g., 8‐OHdG, MDA). Logistic regression showed that each 5 µg/m^3^ increase in second‐trimester PM2.5 was associated with a 1.50‐fold increased risk of preterm birth and a 1.44‐fold increased risk of gestational diabetes after adjustment for maternal age, BMI, WBC, income, and education.

**Conclusion**: PM2.5 exposure during pregnancy is associated with altered urinary lipid profiles, suggesting oxidative stress‐related disruptions in glycerolipid metabolism. These lipidomic changes may mediate the link between PM exposure and pregnancy complications, providing mechanistic insight and potential biomarkers for future risk assessment.

**9:00 AM  –  9:15 AM**

## 78 | Contribution **of Antibody Subtypes to Breastmilk Immunity Against COVID‐19 in Disease and Vaccine Exposed Pregnancies**

Mymy Nguyen^1^; Elke S. Bergmann‐Leitner^2^; Julie A. Jones^1^; Gregory Gromowski^2^; Zubair H. Aghai^3^; Rupsa C. Boelig^4^

^1^Thomas Jefferson University Hospital, Philadelphia, PA; ^2^Walter Reed Army Institute of Research, Silver Spring, MD; ^3^Nemours Children's Health, Philadelphia, PA; ^4^Sidney Kimmel Medical College, Thomas Jefferson University, Philadelphia, PA

**Objective**: To evaluate breastmilk antibody (Ab) subtypes associated with SARS‐CoV‐2 neutralization capacity of breastmilk and differences in Ab profile following maternal COVID‐19 infection versus vaccination.

**Study Design**: Breastmilk was collected longitudinally from those with 1) COVID‐19 in pregnancy 2) both doses COVID‐19 mRNA vaccination in pregnancy or 3) at least one vaccine dose postpartum. Serological assays measured IgG, IgM, systemic IgA, and secretory IgA against SARS‐CoV‐2 spike (S) and nucleocapsid (N) antigens. Neutralization titers were reported as the reciprocal of the dilution necessary to achieve 50% inhibition of wild‐type SARS CoV‐2. Linear mixed effects modeling used to identify association between titers, time, and neutralization capacity.

**Results**: The study included *N* = 8 with COVID in pregnancy, *N* = 29 vaccination in pregnancy and *N* = 23 vaccination postpartum. COVID‐19 infection resulted in significantly higher systemic and secretory IgA levels at delivery compared to vaccination in pregnancy (*p* < 0.05, Figure 1). S‐ IgM and N‐ IgG and were strongly associated with neutralization capacity (*p* < 0.001). S‐ and N‐ secretory IgA demonstrate a stronger association with neutralization capacity (*p* = 0.05, 0.07 respectively) than systemic IgA (*p* > 0.10), although with borderline significance. Principal component analysis revealed distinct Ab profiles in COVID‐exposed vs vaccinated cohorts (Figure 2) related to primarily to IgA titers.

**Conclusion**: COVID‐19 infection elicits a more diverse Ab profile compared to vaccination, although both generate enduring mucosal Ab immunity in breastmilk. Secretory IgA is likely an important component of breastmilk immunity against COVID‐19. Maternal vaccines for infant immunity may be optimized by increasing the secretory IgA response elicited.

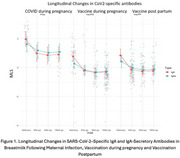

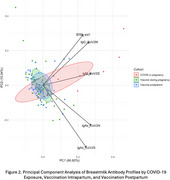

**9:15 AM  –  9:30 AM**

## 79 | **Efficacy of Novel Peptide B7‐33 in Attenuating Preeclampsia Symptoms: Preclinical Study in Rat Models**

Rachel M. Terry^1^; Ahmed F. Pantho^2^; Michelle N. Harris^1^; Kelsey R. Kelso^1^; Niraj Vora^3^; Lorena M. Amaral^4^; Diana Villazana‐Kretzer^3^; Thomas Kuehl^2^; Babbette LaMarca^4^; Mohammad N. Uddin^5^

^1^Baylor Scott & White Health ‐ Baylor College of Medicine, Temple, TX; ^2^Orion Institute for Translational Medicine, Georgetown, TX; ^3^Baylor Scott & White Medical Center, Temple, TX; ^4^The University of Mississippi Medical Center, Jackson, MS; ^5^Texas A&M Health University College of Medicine, Temple, TX

**Objective**: Preeclampsia (PreE) is a hypertensive disorder which affects approximately 10% of gestations. The literature suggests a potential therapeutic role for H2 relaxin in PreE. Due to the complex insulin‐like structure of relaxin (A‐ and B‐ chains), a novel H2 relaxin B‐chain‐only peptide variant, B7‐33, has recently been developed, as well as B7‐33‐Fc, a fusion protein with longer half‐life. This single‐chain peptide displayed equivalent efficacy to the natural H2 relaxin in several functional assays both in vitro and in vivo. The objective of this study is to evaluate whether B7‐33 attenuates PreE effects in rat models of PreE.

**Study Design**: The efficacy of B7‐33 was evaluated in Reduction of Uterine Perfusion Pressure (RUPP) model and desoxycorticosterone (DOCA) model rats. The RUPP rats were randomly assigned to 4 groups (*N* = 8/group): 1) vehicle, 2) B7‐33; 3) B7‐33‐Fc; and 4) B7‐33‐ conjugated with human serum albumin (HSA). Rats were dosed intravenously twice weekly from gestational days (GD) 10 through 20.

The DOCA rats were injected intraperitoneally initially with DOCA, and drinking water was replaced with 0.9% saline. Three groups of animals were studied (*n* = 8/group): 1) normal pregnant (NP) rats; 2) rats administered DOCA and whose drinking water was replaced with NS; 3) rats administered DOCA and saline as for Group 2, and given B733‐Fc intraperitoneally biweekly from GD 10‐20. Mean arterial blood pressure (MAP), proteinuria and inflammatory markers were evaluated. Statistical comparisons were performed using analysis of variance with Duncan's post hoc test.

**Results**: RUPP rats had increased MAP, plasma TNF‐α, and plasma sFlt‐1 along with decreased Nitric Oxide (NO) index compared to NP rats. Treatment with B7‐33 and B7‐33Fc proteins lowered MAP, TNF‐α, and sFlt‐1 to normal levels. B7‐33‐Fc normalizes BP, proteinuria and inflammatory markers in the DOCA rat model of PreE.

**Conclusion**: Both B7‐33 and B7‐33 fusion proteins attenuated the PreE syndrome in RUPP and DOCA rat models of PreE. We concluded that B7‐33 is an ideal candidate as a novel therapy for PreE.

**9:30 AM  –  9:45 AM**

## 80 | Prospective **Evaluation of Platelet Hyperreactivity and Risk of Ischemic Placental Disease**

Christina A. Penfield^1^; Andre A. Robinson^1^; Anais Hausvater^2^; Ariel Schapp^3^; Elliot Luttrell‐Williams^2^; Madison S. House^4^; Yuhe Xia^5^; Valeryia Avtushka^1^; Lauren Kennedy^2^; Rafid Quadir^2^; Justin S. Brandt^6^; Gwendolyn P. Quinn^7^; Ashley S. Roman^6^; Dana R. Gossett^7^; Jeffrey S. Berger^2^

^1^Division of Maternal‐Fetal Medicine, Department of Obstetrics and Gynecology, NYU Grossman School of Medicine, New York City, NY; ^2^Leon H. Charney Division of Cardiology, Department of Medicine, NYU Grossman School of Medicine, New York City, NY; ^3^Division of Hematology Department of Medicine, NYU Grossman School of Medicine, New York City, NY; ^4^NYU Grossman School of Medicine, New York City, NY; ^5^Division of Biostatistics, NYU Grossman School of Medicine, New York City, NY; ^6^Division of Maternal‐Fetal Medicine, Department of Obstetrics and Gynecology, NYU Grossman School of Medicine, New York, NY; ^7^Department of Obstetrics and Gynecology, NYU Grossman School of Medicine, New York City, NY

**Objective**: In addition to their role in thrombosis and hemostasis, platelets are hypothesized to play a pathogenic role in ischemic placental disease (IPD). In vitro demonstration of platelet hyperreactivity is associated with inflammation and cardiovascular risk in nonpregnant populations, but not yet investigated in association with placental health. We prospectively evaluated first trimester platelet hyperreactivity and subsequent development of IPD.

**Study Design: P**regnancy **O**utcomes and **P**latelet **P**henot**Y**pes (POPPY) was a prospective cohort with blood collection at 11‐14 weeks. No participants were on aspirin prophylaxis at the time of blood draw. Platelet function was measured via light transmission aggregometry (LTA) and in vitro platelet hyperreactivity was defined as aggregation >60% with 0.4 uM epinephrine. The primary endpoint was the development of IPD during pregnancy, including hypertensive disorders of pregnancy (HDP), placental abruption, stillbirth, and small for gestational age (SGA) birth. Multivariable logistic regression analysis with adjustment for age, race and/or ethnicity and body mass index was used to estimate likelihood of IPD by platelet hyperreactivity.

**Results**: Among 66 pregnant participants recruited, 61 had LTA evaluation for platelet hyperreactivity in the first trimester. There was a 30.3% prevalence of platelet hyperreactivity (Table). There was a significantly higher risk of IPD in those with hyperreactive platelets at baseline (55% versus 24% in participants without platelet hyperreactivity, *p* = 0.038; Figure). In addition, those with platelet hyperreactivity were more likely to develop individual components of the IPD outcome. After multivariable adjustment, platelet hyperreactivity remained associated with higher odds of IPD (aOR 3.77, 95% CI [1.05‐13.53], *p* = 0.04).

**Conclusion**: Platelet hyperreactivity was associated with a greater than 3‐fold increased risk of IPD. This novel finding implicates platelets in the pathogenesis of placental ischemia and underscores the need for further investigation to elucidate underlying mechanisms and to evaluate the impact of low‐dose aspirin.

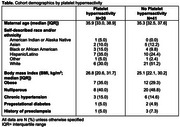

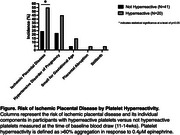

**9:45 AM  –  10:00 AM**

## 81 | **Maternal** and **Fetal Cell Dynamics in the Placenta in Complicated and Healthy Pregnancy**

Rachelly Normand^1^; Paola A. Lopez‐Zapana^2^; Daehee Han^3^; Courtney Ambrose^4^; Roya Best^5^; Zhaojing Liu^2^; Elizabeth Tuttle^5^; Christopher Stueber^5^; Olyvia J. Jasset^2^; Laura Ibanez‐Pintor^2^; Caroline Bald^2^; Lydia L. Shook^6^; Douglas A. Lauffenburger^7^; Alexandra‐Chloé Villani^8^; Andrea G. Edlow^9^

^1^Massachusetts General Hospital, Massachusetts General Hospital, MA; ^2^Vincent Center for Reproductive Biology, Massachusetts General Hospital, Boston, MA; ^3^Vincent Center for Reproductive Biology, Massachusetts General Hospital; Department of Obstetrics and Gynecology, MassGeneral Brigham; Division of Maternal‐Fetal Medicine; Harvard Medical School, Boston, MA; ^4^Center for Immunology and Inflammatory Diseases, Department of Medicine and Krantz Family Center for Cancer Research, Massachusetts General Hospital, Boston, MA; ^5^Massachusetts General Hospital, Boston, MA; ^6^Department of Obstetrics and Gynecology, MassGeneral Brigham; Division of Maternal‐Fetal Medicine; Harvard Medical School, Massachusetts General Hospital/Boston, MA; ^7^Department of Biological Engineering, Massachusetts Institute of Technology, Cambridge, MA; ^8^Center for Immunology and Inflammatory Diseases, Krantz Family Center for Cancer Research, Massachusetts General Hospital, Boston, MA; ^9^Department of Obstetrics and Gynecology, MassGeneral Brigham; Division of Maternal‐Fetal Medicine; Harvard Medical School, Boston, MA

**Objective**: Understanding complex cellular dynamics that govern tolerance and critical placental biology in healthy and complicated pregnancy is key to developing more effective therapeutic strategies. We created a maternal‐fetal cellular atlas of the placenta to characterize common immune‐mediated pregnancy complications.

**Study Design**: We collected 69 placenta samples from patients with a healthy pregnancy (full‐term unlabored *n* = 9, full‐term labored *n* = 9), unlabored indicated preterm birth (vasa previa *n* = 4), and pregnancy complications including spontaneous preterm birth (SPTB *n* = 10), fetal growth restriction (FGR *n* = 9), pre‐term preeclampsia (prePE *n* = 10), term preeclampsia (termPE *n* = 8) and type 1 diabetes (T1D *n* = 10). We profiled samples via multi‐modal10x Genomics single cell sequencing: gene expression, CITE‐seq and paired T cell receptor, with spatial transcriptomics (Visium HD) in a subset for validation. Genetic demultiplexing determined maternal or fetal origin of each cell.

**Results**: We profiled over 1.2 million placental cells, the largest and most diverse atlas to date. We identified 114 cell populations (Figure 1), including never‐described placental subsets of Hofbauer cells (HBCs), PAMMs, trophoblasts, dendritic cells, and ɣδT cell subsets. Across complications, we found more abundant maternal macrophages and decreased HBCs, most notably in prePE and termPE (Figure 2). We identified condition‐specific cellular shifts, such as an increase in maternal NKT cells, a novel HBC population and a ɣδT cell population in SPTB, and reduction in fetal NK cells coupled with an increase in maternal NK and Treg cells in PET. TCR repertoire analysis identified enrichment of expanded clones in maternal T cell subsets.

**Conclusion**: This comprehensive atlas redefines the placental cellular landscape. Decreased fetal and increased maternal placental cell subsets characterized common pregnancy complications, indicating a vital role for maternal‐fetal immunologic balance. These and other mechanistic insights into SPTB, prePE and termPE, FGR and T1D are urgently needed to guide targeted therapeutics.